# Mesoporous SiC-Based Photocatalytic Membranes and Coatings for Water Treatment

**DOI:** 10.3390/membranes13070672

**Published:** 2023-07-16

**Authors:** Karla Begonia Cervantes-Diaz, Martin Drobek, Anne Julbe, André Ayral, Julien Cambedouzou

**Affiliations:** Institut Européen des Membranes, IEM–UMR 5635, Univ Montpellier, CNRS, ENSCM, 34095 Montpellier, France; kbegonia.d@gmail.com (K.B.C.-D.); martin.drobek@umontpellier.fr (M.D.); anne.julbe@umontpellier.fr (A.J.); andre.ayral@umontpellier.fr (A.A.)

**Keywords:** silicon carbide, mesoporous membrane, photocatalysis, membrane reactor

## Abstract

Photocatalytically active silicon carbide (SiC)-based mesoporous layers (pore sizes between 5 and 30 nm) were synthesized from preceramic polymers (polymer-derived ceramic route) on the surface and inside the pores of conventional macroporous α-alumina supports. The hybrid membrane system obtained, coupling the separation and photocatalytical properties of SiC thin films, was characterized by different static and dynamic techniques, including gas and liquid permeation measurements. The photocatalytic activity was evaluated by considering the degradation efficiency of a model organic pollutant (methylene blue, MB) under UV light irradiation in both diffusion and permeation modes using SiC-coated macroporous supports. Specific degradation rates of 1.58 × 10^−8^ mol s^−1^ m^−2^ and 7.5 × 10^−9^ mol s^−1^ m^−2^ were obtained in diffusion and permeation modes, respectively. The performance of the new SiC/α-Al_2_O_3_ materials compares favorably to conventional TiO_2_-based photocatalytic membranes, taking advantage of the attractive physicochemical properties of SiC. The developed synthesis strategy yielded original photocatalytic SiC/α-Al_2_O_3_ composites with the possibility to couple the ultrafiltration SiC membrane top-layer with the SiC-functionalized (photocatalytic) macroporous support. Such SiC-based materials and their rational associations on porous supports offer promising potential for the development of efficient photocatalytic membrane reactors and contactors for the continuous treatment of polluted waters.

## 1. Introduction

Water is considered as one of the most important natural resources for life on our planet. Unfortunately, not all the fresh water available is drinkable because most of the drinking water sources are polluted with heavy metals, pathogens, fertilizers, and pesticides, mainly coming from industrial wastes discharged into nature without any treatment [1]. Among the main pollutants today, we can mention in particular the dyes produced for the textile industry, which consume large quantities of water and pollute water bodies with mutagenic and toxic substances [2].

Many techniques are commonly used to treat contaminated waters, e.g., coagulation, flocculation, ozonation [3], etc. However, their efficiencies are often questioned due to the possible production of undesirable, or even more toxic and difficult to handle, secondary by-products [4].

One of the promising strategies to overcome this drawback is based on the application of efficient photocatalytic materials, as detailed elsewhere [5,6,7,8,9]. Moreover, membrane systems ensuring the elimination of water pollutants by filtration processes, possibly coupled with photocatalytic reactions induced at the membrane–liquid interface, represent another relevant option studied in the literature [10]. In such hybrid systems combining photocatalysis with pressure-driven membrane processes, e.g., ultrafiltration [11] and nanofiltration [12], different possible configurations can be considered. It is typically possible to implement thin layers of active membranes (micro/mesoporous) on the upper surface of an asymmetric macroporous ceramic support. Indeed, in addition to their molecular cut-off determined by the material pore-size, if photo-catalytically active, they can also be used to overcome the fouling of membrane by adsorbed organic pollutants [13]. Another approach consists of a surface functionalization of the grains of the porous asymmetric ceramic support (permeate side) with a photo-catalytically active material. Such a system configuration ensures a mineralization of the remaining (smaller) organic molecules in the permeate, which could have passed through the membrane filtration layer [14].

The majority of photocatalytic membrane reactors described in the literature are based on an application of titanium dioxide (TiO_2_) [15,16,17,18] mainly in an anatase crystalline form due to its well-known photoactivity under UV irradiation (bandgap for TiO_2_ anatase is 3.2 eV). TiO_2_ is usually deposited as thin films supported on ceramic membrane substrates (e.g., α-alumina) [16,17].

In an effort to explore the potential application of other types of photoactive materials, this work was focused on composite layers based on silicon carbide (SiC) due to their attractive physico-chemical properties (including, namely, abrasion resistance) possibly exceeding those of TiO_2_ [19]. The photocatalytic properties of SiC already attracted tangible attention in recent literature [19,20,21,22,23,24]. This material typically features a wide bandgap [25], which can, however, vary depending on the SiC polytype (cubic, hexagonal). For example, 2H-SiC and 3C-SiC exhibit an indirect bandgap reaching values of 3.33 eV and 2.52 eV, respectively [26]. On the contrary, the direct bandgap has been observed for β-SiC in the form of nanowires [27]. SiC hollow spheres with a bandgap of 2.4 eV were used for the photocatalytic reduction of CO_2_ [24], whereas another study proposed SiC (bandgap of 2.5 eV) for the water splitting reaction [28]. Finally, SiC nanowires [29] and hollow SiC spheres [30] with smaller bandgaps (2.35 eV and 2.15 eV, respectively) were found to show high photocatalytic activities for hydrogen production or dye degradation under visible light irradiation. Such SiC reactivity for dye degradation under visible light irradiation was also recently demonstrated for SiC foams [31].

Considering the application of SiC as selective membrane layers, many studies were also reported by different research groups due to the vast potential of this material for both liquid and gas phase separations [32]. Indeed, SiC membranes are attractive due to their both high physicochemical stabilities [20] and specific surface properties (naturally and permanently hydrophilic) [21,22], inducing high water permeability compared to other ceramic and polymeric counterparts [20]. A huge challenge today lies in the control of their microstructure and, more specifically, in the development of mesoporous SiC membrane layers, still scarcely investigated in the literature.

In such a context, this work aims to highlight the possibility of using original photocatalytic SiC/α-Al_2_O_3_ composites by coupling an ultrafiltration SiC membrane top-layer with a SiC-functionalized (photocatalytic) macroporous support. This functional SiC material featuring superior physicochemical properties compared to other photocatalysts (e.g., semiconducting metal oxides) is expected to offer great potential for application as a membrane reactor for the continuous treatment of contaminated wastewater in ambient conditions. Photoactive SiC-based composite materials have been prepared in the form of thin layers deposited on carbon-modified macroporous α-alumina supports. The preparation is based on the so-called preceramic polymer route, in which polymer precursors can be converted to SiC by high temperature pyrolysis (>1000 °C) in argon (Ar) atmosphere.

**Figure 1:**
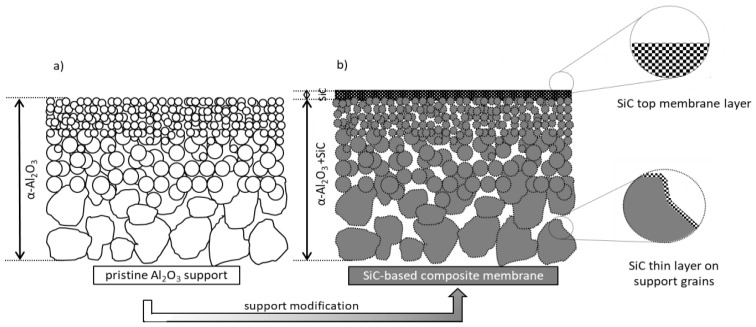
Schematic representation of (**a**) the pristine asymmetric membrane support and (**b**) the final design of the new photocatalytic SiC-based membrane system.

The intended design (Figure 1) consists of a continuous mesoporous layer (membrane) of photoactive SiC on the upper surface of the support, whereas a SiC coating of the same material covers the support grain surface, thereby offering functional photocatalytic properties at the permeate side.

The main advantage of such a concept bears a possible coupling of the separation capacity of the SiC mesoporous top-layer (permselective membrane) with the photocatalytic activity of the SiC material. Such photocatalytic activity might be used for the mitigation of membrane fouling and/or the degradation of small molecules not retained by the membrane. Hence, the two configurations of hybrid membrane systems described previously (i.e., photocatalytic membrane at the feed side and/or photocatalytic support at the permeate side) are assumed to be accessible simultaneously by implementing a simple and robust method for the functionalization of commercially available asymmetric ceramic supports with SiC membrane (top-layer) and coatings (on support grains).

In order to properly describe the membranes and the as-prepared coated support systems, the chemical and structural characterizations of the deposited SiC-based layers wree carried out in parallel with the evaluation of the membrane’s performance in terms of permeability and selectivity. The photocatalytic activities of the coated/functionalized supports were then evaluated by considering their efficiencies for the degradation of a model molecule (methylene blue, MB) under UV light irradiation. The specific degradation rate was recorded both in diffusion mode and in permeation mode, allowing us to assess the catalytic performance of the new SiC/α-Al_2_O_3_ composite material compared to TiO_2_-based membranes.

## 2. Materials and Methods

### 2.1. Materials

Two geometries (flat and tubular) of α-Al_2_O_3_ ceramic membrane supports were used in this study. The flat disks (Ø = 26 mm, thickness =1 mm) were supplied by Fraunhofer IKTS (Fraunhofer Institute for Ceramic Technologies and Systems, Hermsdorf, Germany), whereas the tubular membrane supports (ID/OD = 10/7 mm; total length = 50 mm) were supplied by Pall-Exekia, France. Both supports exhibited an asymmetric structure, with a top membrane layer having a nominal pore size of 200 nm, intermediate layers with pore sizes of several hundred nm, and a macroporous support with pore sizes of 10 µm for tubes and 3 µm for flat support geometries, respectively. Before SiC deposition, support modification was carried out with D-(+)-Glucose (C_6_H_12_O_6_)–anhydrous (96%), obtained from Sigma-Aldrich. SiC was synthesized from allylhydridopolycarbosilane (AHPCS) precursor with the structural formula (SiH_2_CH_2_)_0.9_(Si(allyl)HCH_2_)_0.1_, purchased from Starfire Systems under the trade name SMP-10. Polystyrene-block-polybutadiene-block-polystyrene (SBS) block copolymer (30 wt%, MW = 140) purchased from Sigma-Aldrich was used as a porogen. Cyclohexane C_6_H_12_ (ACS 99+%) used as solvent was obtained from Alfa Aesar. For the filtration and photocatalytic tests, the methylene blue dye (C_16_H_18_ClN_3_S_x_H_2_O) purchased from Sigma-Aldrich was used as a model pollutant.

### 2.2. Support Treatment and Membrane Preparation

Before any utilization, the tubular ceramic supports were previously sealed at their extremities with a high temperature commercial glaze (deposited over 1 cm in length, in/out) in order to avoid any leakage during gas and water permeation tests. These enameled supports were used to evaluate the quality of the SiC thin layers and their pore sizes by both single gas permeance and clean water permeability measurements.

Before deposition of the SiC membrane on the α-Al_2_O_3_ supports (tubes and discs), a carbon buffer layer was first deposited by following a protocol adapted from Madsen et al. [33], based on the pyrolysis of glucose. The objective was to avoid the formation of an oxide material at the interface between the SiC layer and the α-Al_2_O_3_ support. The solution for the deposition of the SiC material on the carbon-coated α-Al_2_O_3_ supports was prepared by dissolution of the SBS porogen in cyclohexane under continuous stirring at room temperature. Finally, AHPCS was added dropwise to the SBS solution under stirring, thus yielding a perfectly homogeneous AHPCS-SBS solution of light-yellowish color. The molar ratio between AHPCS and SBS was set at 1:1.

The carbon coated α-alumina supports were immersed into the as-prepared AHPCS-SBS solution and maintained under vacuum (700 mmHg) for 15 min. This coating step was followed by the pyrolysis of the entire composite membrane in a tubular furnace (heating rate 120 °C/h to 1200 °C, 2 h dwell at 1200 °C) under an argon atmosphere.

The tubular membranes (previously sealed with enamel) were tested directly, whereas the flat disk membranes were sealed afterwards with a silicone rubber on their outer diameters. The as-prepared membranes were tested regarding the filtration ability of the SiC top-layer and the photocatalytic efficiency of the functional SiC layer covering the support grains. For the latter tests, due to a total retention of methylene blue by the filtration layer (discussed later), the SiC top layer as well as the upper and intermediate layers of the functionalized asymmetric flat disks were scraped off to obtain only a symmetric macroporous filter with its coarse alumina grains coated with a thin layer of SiC.

It should be noted that equivalent powders were synthesized by pouring the identical solution used for the SiC–membrane synthesis into wide ceramic crucibles for some specific physico-chemical characterizations (e.g., N_2_ physisorption analysis, XRD, FTIR), followed by the same thermal treatment as described above. Note that the alumina crucibles were previously coated with a carbon layer (quantity and synthesis method mimicking the membrane support modification).

### 2.3. Materials Characterization

XRD experiments were performed on a Panalytical Xpert diffractometer using CuKα radiation as the X-ray source. FTIR was performed on a Nexus spectrometer in the range [650–4000 cm^−1^].

The morphologies of the as-prepared SiC-based membranes were examined with a Hitachi S4800 scanning electron microscope (SEM), whereas element composition was determined by energy-dispersive X-ray spectroscopy (EDX) at using Zeiss EVO HD15 SEM coupled to an Oxford Instruments X-Max SDD detector. Nitrogen sorption measurements were performed on the Micrometrics ASAP 2020 instrument. The specific surface area was obtained by means of the BET model, and the pore size distributions were calculated by applying the Barret–Joyner–Halenda (BJH) method. The reflectance of the studied membranes was measured by UV-visible spectrophotometry using a Perkin Elmer Lambda spectrometer equipped with an integrating sphere (150 mm in diameter). The hemispherical reflectance spectrum was obtained for wavelengths ranging from 250 to 2500 nm. The optical bandgap was calculated from the reflectance values using the Kubelka–Munk function [34]. The dynamic characterization tests of the membranes’ quality and the evaluations of their pore sizes were carried out only with SiC membranes synthesized on tubular supports, which offered higher mechanical resistances. Indeed, unlike disks that tended to break when exposed to high transmembrane pressures, tubular supports were significantly more robust. Gas permeation experiments were carried out with nitrogen and helium in a home-made gas experimental setup, as described in a previous work [35]. All the measurements were performed at room temperature and transmembrane pressures up to ΔP = 1 bar. Water permeability tests were carried out using a similar permeation cell connected to a stainless-steel tank with deionized water. Permeation measurements were performed by constant pressurization of water with N_2_ gas at ΔP ~ 11 bars on the feed side of the membrane. Water permeability was measured by collecting the liquid on the permeate side at defined transmembrane pressures.

### 2.4. Evaluation of MB Removal in Diffusion Mode Configuration

**Figure 2:**
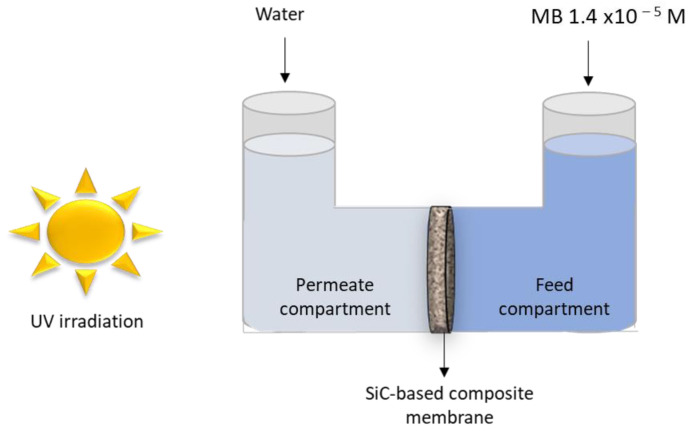
Schematic of the experimental setup for evaluation of MB removal in diffusion mode.

The performance of the SiC/α-Al_2_O_3_ composite material for MB removal was evaluated using the experimental setup shown in Figure 2 and following a similar procedure developed in our previous work [36]. The setup consisted of a diffusion cell with two borosilicate compartments separated by the functionalized support (flat disk geometry) sealed with two rubber rings that delimited an active surface area of 3.14 × 10^−4^ m^2^. These experiments were carried out after abrasion of the SiC top-layer and all the intermediate layers of the SiC-functionalized disk in order to minimize the adsorption effect of the SiC material on MB removal. It is worth mentioning that the adsorption capacity of SiC has already been investigated in our previous works [31]. The abraded side of the resulting symmetric macroporous filter faced the feed side of the diffusion cell, whereas the other side of the support (with support grains coated with SiC) faced the permeate side. The feed cell compartment was filled with 80 mL of 1.4 × 10^−5^ M MB solution, whereas the permeate side of the diffusion cell contained 90 mL of distilled water. Before starting the diffusion measurements, the functionalized supports were saturated by MB. Both sides of the cell were kept under continuous stirring at room temperature.

For UV irradiation of the permeate side of the diffusion cell, the radiation source applied was a CLEO compact UV lamp with an emission spectrum having a maximum peak at λ ~ 350 nm. The irradiance (37 W/m^2^) was measured with a UV radiometer (EIT UVICURE UV radiometer) placed at the same distance of the lamp as the irradiated support side of the disk.

The photocatalytic experiments were carried out during intermittent periods of one hour, with a typical duration of 8 h for each measurement, comprising three periods of UV irradiation (3 × 1 h).

For the sake of comparison, blank sample experiments were conducted with the pristine α-Al_2_O_3_ support to verify the absence of photolytic degradation of MB and thus ensure that the decrease in MB concentration was exclusively caused by photocatalytic effect of the SiC material. As with the SiC-functionalized disks, the top and intermediate layers were polished with the abraded side facing the feed compartments of the cells. The degradation rate was calculated systematically for each experiment. An aliquot volume was taken every hour to measure its absorbance (at λ = 664 nm) by the UV-vis spectrophotometer to record the MB concentration (i.e., MB degradation kinetics) over time. It should be noted that the initial concentration of MB on the permeate side of the diffusion cell was zero because it contained only pure water at the start of the experiment.

### 2.5. Evaluation of MB Removal in Permeation Mode Configuration

**Figure 3:**
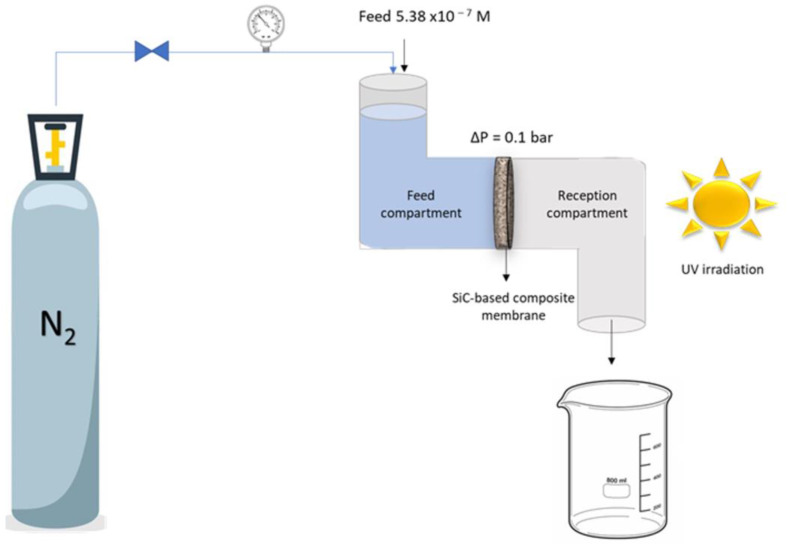
Schematic of the experimental setup for evaluation of MB removal in permeation mode.

The experimental setup for photocatalytic tests performed in permeation mode configuration (Figure 3) was adapted from the one used previously (in diffusion mode). The feed side of the permeation cell was filled with MB at a concentration of 5.4 × 10^−7^ M. The permeate side was opened to the atmosphere, enabling a continuous collection of the permeating liquid. As previously, the experiments were carried out with abraded disks with the abraded side facing the feed side compartment.

The first series of experiments was performed in the absence of any light irradiation. A transmembrane pressure of ΔP = 0.1 bar, applied to the liquid on the feed side, was generated by pressurized N_2_. The liquid was collected on the permeate side, and the concentration of MB was measured with a UV-vis spectrophotometer, as described previously.

The second series of experiments was carried out in the presence of UV light irradiation applied to the permeate compartment of the cell. The solution passing through the functionalized disk was collected, and the MB concentration was determined with a spectrophotometer.

## 3. Results and Discussion

### 3.1. Material Characterization

A series of physico-chemical characterizations has been carried out to determine the structural and textural properties of the prepared SiC-based materials. For technical reasons (too low quantity of SiC material on ceramic supports), some analyses, e.g., XRD, IR, or N_2_ physisorption measurements, had to be conducted on equivalent powders of the same composition, prepared as detailed in the experimental part. A SEM image of the equivalent SiC powder is shown in Supplementary Information (S1).

**Figure 4:**
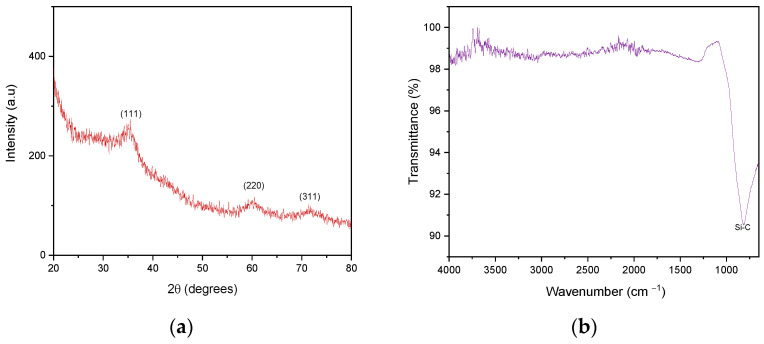
(**a**) XRD pattern (λ = 1.54 Å) and (**b**) FTIR spectrum of the SiC-based powder.

Figure 4a shows the XRD pattern of the powdered SiC-based material with low crystallinity corresponding to the SiC coated on the α-Al_2_O_3_ support. The Bragg peaks at 2θ = 35.5, 60.7, and 71.3° fit the (111), (220), and (311) lines of the cubic SiC, albeit with an amorphous character linked to the low synthesis temperature, as described elsewhere [37,38]. The nature of the material has been further confirmed by FTIR analysis (Figure 4b) detecting the well-defined Si-C absorption band near 800 cm^−1^ in good agreement with the results obtained in our previous work, thus confirming the presence of the SiC network [38].

**Figure 5:**
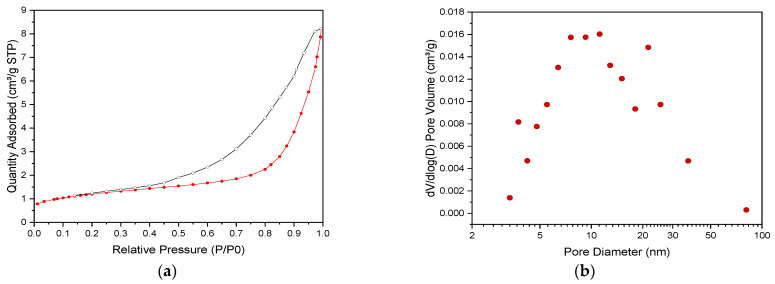
(**a**) Nitrogen adsorption–desorption isotherms at 77 K of equivalent SiC-based powder sample (red: adsorption branch, black: desorption branch) and (**b**) pore size distribution according to the BJH method (desorption branch).

The N_2_ physisorption experiments with the equivalent SiC powder revealed type IV adsorption–desorption isotherms according to the IUPAC classification (Figure 5a), thus confirming the presence of mesopores in the material. Figure 5b shows the BJH pore size distribution, which appears to be quite large with a maximum of the distribution corresponding to mesopores ~ 10 nm in diameter. It should be noted that the material featured a relatively small specific surface area: the values calculated by using the BET method never exceeded 5 m^2^/g.

### 3.2. Membrane Characterisation

#### 3.2.1. Membrane Morphology

**Figure 6:**
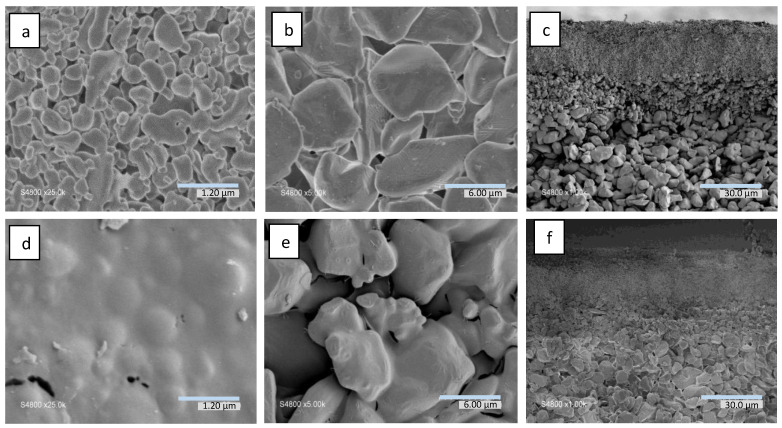
SEM images of (**a**) top surface, (**b**) bottom surface, (**c**) overall cross-section of pristine α-Al_2_O_3_ disk, (**d**) top surface, (**e**) bottom surface, and (**f**) overall cross-section of SiC-based composite membrane on α-Al_2_O_3_ disk.

The morphologies of both the SiC-based composite membranes and the pristine α-Al_2_O_3_ supports (cross-section, upper and bottom layers) were observed by SEM (Figure 6). Comparison of the virgin α-Al_2_O_3_ support (Figure 6a–c) and the SiC-based membrane (Figure 6d–f) allowed us to distinguish a thin layer formed on the top surface of the support (pore size ~ 200 nm) after SiC deposition. On the opposite side of the asymmetric support (pore size ~ 10 μm, Figure 6d,e), a thin layer of SiC homogeneously covering the α-alumina grains could be clearly observed. It should be underlined that this functional SiC layer covering the surface of the grains did not obstruct the pores of the support, thus having practically no impact on its primitive hydraulic resistance. Energy-dispersive X-ray (EDX) analysis performed in different areas of the membrane evidenced the highest amount of Si at the top and bottom of the membrane, with 6.4 at.% and 4.5 at.% of Si, respectively (Supplementary Information S2). On the other hand, at the very center of the support cross-section, Si could hardly be detected, thus indicating an inhomogeneous impregnation in the support bulk, with the SiC deposit mainly located on the most external parts of the support. Considering that the photoactive SiC coating must be accessible for light irradiation, it must be located on the external parts of the support and thus the limited infiltration of SiC inside the support did not present any drawback for the photocatalytic performance of the composite system. It could be noted that the C/Si atomic ratio on both the top and bottom sides of the membrane was in the range ~3–4 due to the pre-treatment of the alumina support with a carbon layer, thus resulting in a carbon-rich, SiC-based composite material. It should be underlined that the EDX analysis carried out on the equivalent SiC-based powders (S1) revealed an almost equal C/Si atomic ratio, thus confirming their same chemical composition as the SiC-based layers deposited on α-Al_2_O_3_ supports.

#### 3.2.2. Gas Permeability Measurements

The permeance of single gases (He and N_2_) through the SiC-based membranes deposited on α-Al_2_O_3_ enameled tubular supports was measured at 25 °C and ΔP = 1 bar. The objective was only to confirm the continuity of the SiC membrane top-layer and obtain an indirect evaluation of its mean pore size [39,40,41].

The permeance values measured for N_2_ and He were 3.6 × 10^−6^ and 8.3 × 10^−6^ mol/(m^2^sPa), respectively. No increase in the permeance was detected while increasing the applied transmembrane pressure up to from 0.25 to 1 bar (see S3 in Supplementary Information), suggesting a limited contribution of viscous flow and thus revealing the absence of large interconnected macroporous defects. This finding confirmed the formation of a continuous SiC top layer uniformly covering the 200 nm pores of the support, as already observed by SEM (Figure 6d). The membrane’s permselectivity for He/N_2_ gas pairs was ~2.35, a value approaching the Knudsen selectivity (α_K_(He/N_2_) = 2.64) [42], characteristic for mesoporous membrane materials.

#### 3.2.3. Water Permeability Measurements

In order to further confirm the mesoporous character of the membrane material and to estimate the molecular cut-off of the membrane top-layer, water permeability measurements were carried out with deionized water, using the same tubular membrane previously used for gas permeation measurements. The membrane pore size was estimated by applying the Kozeny–Carman Equation (1), which assumes that pores are voids between spherical particles:

(1) $$J=\frac{{∆P\;\varepsilon }^{3}}{K\mu S^{2}{\left(1-\varepsilon \right)}^{2}\;L}$$

with *J* as the flux (m^3^/m^2^.s), *S* as the particle specific surface area (m^2^/m^3^), ∆P as the pressure drop (Pa), *L* as the membrane thickness (m), μ as the water viscosity (Pa.s), ε as the porosity, and *K* as the empirical Kozeny–Carman constant.

Assuming that the grains forming the porous structure of the membrane are ideal spheres, the grain radius (*r*) could be calculated with the following Equation (2):

(2) $$r=\frac{3}{S}\;$$

Water permeation results with calculated pore radii are presented on Table 1. Water fluxes were found to be stable at the applied trans-membrane pressures and did not change over time (measurements made over several hours). The calculated grain diameters (d = 2*r*) were of the order of ~33 nm. Considering a compact packing of non-aggregated spherical particles, which generated pores whose diameters were about one-third of the diameter of the particle itself, the average pore diameter in the membrane material was expected to be close to 10 nm. Of course, the absolute values of the calculated pore diameter should be taken with caution because the calculation applied to a model membrane material. However, the estimated value of pore size perfectly fit the results derived from the N_2_ adsorption–desorption analysis of the equivalent powder.

Hence, it could be assessed that the applied synthesis protocol led to the formation of a mesoporous SiC separation layer with promising potential in applications targeting ultrafiltration processes.

### 3.3. Photocatalytic Activity

The SiC/α-Al_2_O_3_ composite membrane has been first used as-prepared to evaluate the semiconducting properties of the deposited SiC material.

In a second step, the photocatalytic efficiency of the SiC material for the elimination of MB has been investigated in both diffusion and continuous mode configuration. In order to evaluate the actual photocatalytic activity of the functional coating on the support grains, the mesoporous SiC top-layer has been removed for these tests. Indeed, the strong adsorption capacity of the mesoporous membrane and its separation efficiency makes it problematic to access the actual photocatalytic properties of the functional system due to the lack of MB molecules to degrade on the permeate side of the diffusion cell. Consequently, for this series of tests, the SiC membrane top-layer (facing the feed side of the diffusion cell) has been removed by mechanical abrasion. The tested material was thus a macroporous catalytic contactor with high permeability, in which the photoactive SiC catalyst covers the grains of the α-Al_2_O_3_ macroporous support. This procedure should not negatively impact the catalytic performance of the system, as only the opposite side of the membrane (governing the photocatalytic reaction) is subjected to direct light irradiation.

#### 3.3.1. Electronic Characterization of the SiC-Based Layer

**Figure 7:**
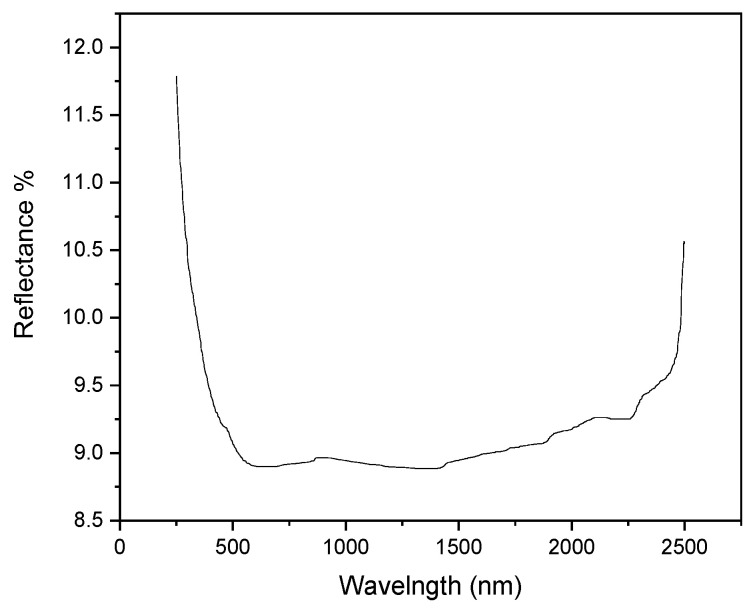
Reflectance spectra of the SiC-based composite membrane.

In order to evaluate the semiconducting properties of the studied SiC material, the reflectance spectra were recorded on SiC supported membranes (Figure 7).

**Figure 8:**
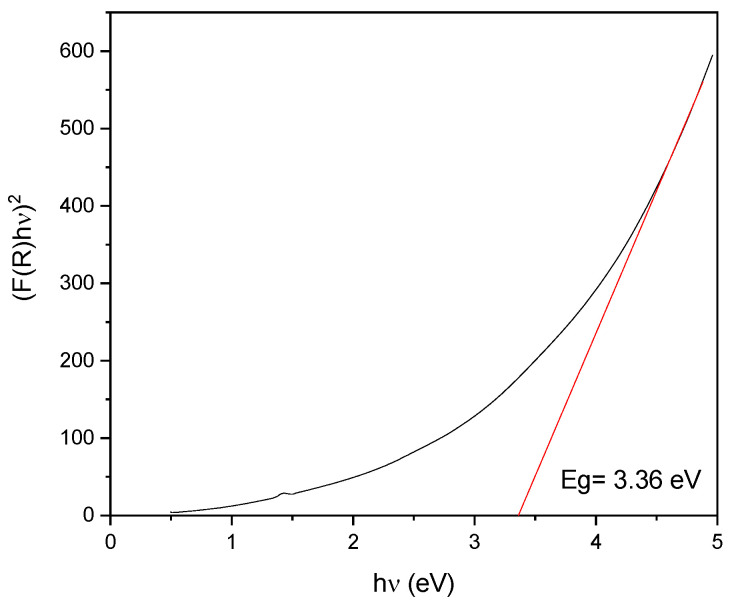
Bandgap calculated for SiC-based supported membrane.

The measurements were performed on the membrane side of the ceramic support (SiC top-layer on SiC/α-Al_2_O_3_). The band gap energy was determined with the Kubelka–Munk function F(R), plotting ${\left(F(R)h\nu \right)}^{1/n}$ (Equation (3)) as a function of photon energy hυ and from the intersection with the *x*-axis of the linear fit of the region associated with the optical absorption edge [43,44,45]. A more detailed development of the function is presented in supplementary information (Supplementary Information S4).

(3) $${\left(F(R)h\nu \right)}^{1/n}=A(h\upsilon -E_{g})$$

with $A\prime ={\left(\frac{2}{S}\right)}^{\frac{1}{n}}A$ as a constant [46] depending on *S* (scattering coefficient), h as Planck’s constant, υ as the photon frequency, *E_g_* as the bandgap energy, and *n* determines the transition nature with values of *n* = 1/2 for direct allowed transition, *n* = 3/2 for direct forbidden transition, *n* = 2 for indirect allowed transition, and *n* = 3 for indirect forbidden transition [43,44,45]. Results of bandgap calculations for the SiC-based supported membranes are shown in Figure 8.

The nature of the transition for the materials was found to be a direct allowed transition, as the best fit was achieved with *n* = 1/2. The bandgap for the SiC-based supported membranes was 3.36 eV (369 nm, in the UV range), a value which corresponded to those typically reported by other authors (between 4.48 eV [47] and 3.05 eV [48]). The decrease in the bandgap for the SiC-modified alumina support thus clearly conferred new semiconducting properties to the as-prepared composite material. For the sake of comparison, band gap measurements were also performed on the pristine α-Al_2_O_3_ support. The reflectance spectrum and bandgap calculations are shown in Supplementary Information (S5). The bandgap was determined at ~4.48 eV, which was rather low value for this type of insulator. On the other hand, considering that this was only a surface analysis on possibly hydrated/hydroxylated α-Al_2_O_3_, the result was well in line with the values reported in the literature for α-Al_2_O_3_ thin films [49].

#### 3.3.2. Evaluation of MB Elimination Efficiency in Diffusion Mode Configuration

**Figure 9:**
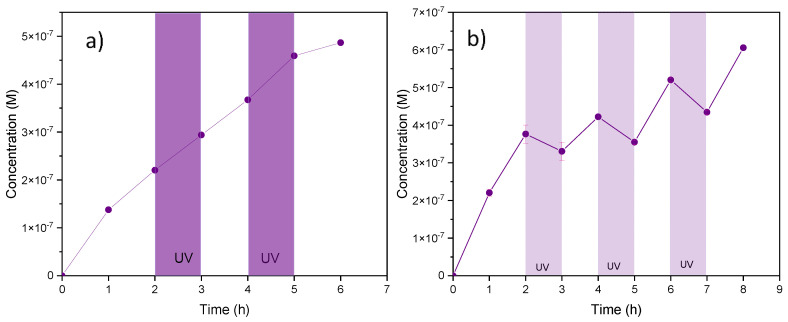
Evolution of MB concentration under UV light irradiation (λ = 350 nm at 37 W/m^2^) in the presence of (**a**) the pristine α-Al_2_O_3_ support and (**b**) the SiC-coated α-Al_2_O_3_ support (functionalized macroporous filter). In all experiments, the initial MB concentration was 1.4 × 10^−5^ M, the pH of the solution was ~6.3, and the active surface of catalytic membrane was delimited to ~3.14 × 10^−4^ m^2^.

The photocatalytic activity of the abraded SiC-based composite (functionalized macroporous filter) was studied by means of the degradation of MB under UV irradiation in the setup described in the experimental part. It should be emphasized that this model molecule was selected to benefit from its full mineralization, which could be easily achieved by the application of semiconductor photocatalysts, as described elsewhere [50]. In the present study, the contribution of photolysis (if any) was assessed using the pristine α-Al_2_O_3_ support for blank experiments. It was confirmed (Figure 9a) that UV irradiation had almost no impact on MB degradation when the pristine α-Al_2_O_3_ support was used in the diffusion cell. On the other hand, it could be seen (Figure 9b) that the MB concentration decreased when the SiC-based composite was used as a photocatalyst under UV light irradiation. Indeed, this observation clearly confirmed the photocatalytic activity of the SiC material because an opposite phenomenon occurred after each period without irradiation, leading to an increase in the MB concentration on the permeate side of the diffusion cell. It should also be noted that the adsorption effect of the support was experimentally confirmed to be negligible. As described elsewhere [51], the photocatalytic activity was induced by the semi-conductor character of SiC forming highly reactive species (i.e., holes, OH, and O_2_^−^) under the effect of UV light irradiation. Each experiment was repeated at least five times at different time periods to assess catalyst stability/reusability.

To quantify the photocatalytic efficiency, the specific degradation rate *δ* was calculated as the quantity of MB eliminated per unit time and membrane surface, according to Equation (4) [14]:

(4) $$\delta =\frac{V\left(C_{WI}-C_{UV}\right)}{t\times A}$$

with *C_UV_* as the MB concentration on the reception side of the photocatalytic cell at the end of the irradiation period (time *t*), *C_WI_* as the MB concentration theoretically reached in the dark (absence of any UV irradiation), *V* as the volume of the solution in reception tank of the photocatalytic cell, and *A* as the active filter area. An example of a graphical representation used to determine the concentration values for the calculation of the specific degradation rate *δ* is shown in Supplementary Information (S6). Furthermore, the cumulative amount of degraded MB and its abatement rate vs. time are shown in Supplementary Information (S7).

For several successive irradiation periods and a series of equivalent disk samples, the average value obtained for *δ* was 1.58 × 10^−8^ mol s^−1^ m^−2^. It should be emphasized that a virtually zero decrease in the specific degradation rate was observed for repeated experiments, thus confirming the stability of the tested photocatalytic material and its potential reuse. In comparison with the literature data (Table 2), this specific degradation rate observed for the studied SiC-functionalized disk was of the same order of magnitude as for conventional TiO_2_ membrane materials. A comparison of degradation performance for selected photocatalytic TiO_2_-based membranes is shown on Table 2. It should be emphasized that even if the performance of the SiC/α-Al_2_O_3_ disk did not exceed those of the TiO_2_ photocatalytic materials, such macroporous SiC-functionalized filters deserved attention for specific application conditions, given their superior physico-chemical properties, particularly in terms of chemical and mechanical stability.

#### 3.3.3. Evaluation of MB Elimination Efficiency in Continuous Mode Configuration

At the start of the experiment, the MB solution was forced to permeate through the SiC/α-Al_2_O_3_ disk in the absence of UV irradiation. The MB concentration was measured regularly until reaching almost zero difference in the absorbance of the MB solution between the feed side and the permeate side of the cell. This observation suggested complete saturation of the SiC coating material with MB molecules, thus excluding any possible additional decrease in MB concentration due to adsorption phenomena. The solution absorbance (MB concentration) decreasing upon irradiation of the disk with UV light (Table 3) could, therefore, be attributed exclusively to the photocatalytic activity of the SiC material. The volume of permeate, the corresponding permeation time, as well as the change in MB concentration were used to calculate the specific degradation rate, using Equation (4) as before.

The average specific degradation rate *δ* was found to be 7.5 ± 0.3 × 10^−9^ mol s^−1^ m^−2^. This value was low compared to that obtained in diffusion mode, due to the shorter contact time at the interface between the liquid and the SiC photocatalytic coating. However, it always remained in the same order of magnitude, thus confirming the interest of this material for the photocatalytic degradation of water pollutants on the permeate side of the SiC-functionalized disk during continuous filtration.

## 4. Conclusions

Photocatalytically active silicon carbide (SiC)-based mesoporous layers have been successfully prepared and deposited on commercial macroporous α-alumina supports. The proposed approach combined preceramic polymer conversion, pore formation by soft-templating, and liquid phase impregnation. Microstructural characterization of the materials revealed the mesoporous character of the SiC layers, whose presences were confirmed by SEM and EDX analyses on both the upper and bottom sides of the α-alumina support. Reflectance spectroscopy measurements of the SiC thin layers determined an optical bandgap of 3.36 eV, confirming their semiconducting properties and thus their potential applicability as photocatalysts under UV light irradiation. The photocatalytic activity was demonstrated by measuring the degradation efficiency of a model organic pollutant (methylene blue, MB) under UV light irradiation in both diffusion and permeation modes using SiC-coated macroporous supports. The performance of the new SiC/α-Al_2_O_3_ materials was found to compare favorably to conventional TiO_2_-based photocatalytic membranes, specifically when considering the superior abrasion resistance of SiC.

The developed synthesis strategy yielded original photocatalytic SiC/α-Al_2_O_3_ composites with the possibility to couple the ultrafiltration SiC membrane top-layer with the SiC-functionalized (photocatalytic) macroporous support. This type of catalytic contactor could represent a real asset for the future development of new decontamination systems (e.g., membrane reactors for the continuous treatment of polluted waters), coupling both membrane filtration and photocatalytic degradation on the feed and/or the permeate side of the filtration element.

## Figures and Tables

**Table 1:** Water permeation measurements and associated grain radii calculations for SiC-based tubular membrane.

ΔP (bar)	Water Flux J (m^3^/m^2^.s)	Grain Surface Area S (m^2^/m^3^)	Grain Radius r (nm)
10.8	7.04 × 10^−6^	1.83 × 10^8^	16.4
11.1	7.34 × 10^−6^	1.82 × 10^8^	16.5
11.1	7.79 × 10^−6^	1.76 × 10^8^	17.0

**Table 2:** Specific degradation rates δ of MB with several titania-based membranes reported in the literature, in comparison with the new SiC-functionalized α-Al_2_O_3_ disk.

Membrane Material	Crystalline Structure of the Active Phase	Specific Degradation Rate *δ* (mol s^−1^ m^−2^)	Reference
Composite membrane with dispersed TiO_2_ powder (P25-Evonik)	Anatase (70–80 wt%) + Rutile (20–30 wt%)	2.0 × 10^−8^	[52]
TiO_2_ derived from PE-CVD deposition	Anatase	2.5 × 10^−8^	[53]
TiO_2_ from commercial hydrosol	Anatase	2.3 × 10^−8^	[36]
Mesostructured TiO_2_	Anatase	10 × 10^−8^	[54]
SiC-based composite: SiC on carbon coated α-alumina	Cubic/poorly crystalline	1.6 × 10^−8^	This work

**Table 3:** Specific degradation rates δ of MB with the SiC-functionalized α-Al_2_O_3_ disk in continuous mode configuration.

Irradiation Time T (s)	Volume of Solution V (ml)	MB Concentration C (M)	Difference in MB Concentration ΔC (M)	Specific Degradation Rate *δ* (mol s^−1^ m^−2^)
0	_____	5.4 × 10^−7^	_____	_____
490	9.9	4.3 × 10^−7^	1.1 × 10^−7^	7.1 × 10^−9^
509	9.9	4.2 × 10^−7^	1.2 × 10^−7^	7.6 × 10^−9^
646	9.7	3.8 × 10^−7^	1.6 × 10^−7^	7.7 × 10^−9^

## Data Availability

The data presented in this study are available on request from the corresponding author. The data are not publicly available due to the Institution policy.

## Supplementary Materials

The following supporting information can be downloaded at: https://www.mdpi.com/article/10.3390/membranes13070672/s1.
