# Supplementary material for: Mesoporous SiC-Based Photocatalytic Membranes and Coatings for Water Treatment
Source: Membranes (Basel). 2023 Jul 16;13(7):672. doi: 10.3390/membranes13070672 (PMC10383672; doi:10.3390/membranes13070672)
Supplement: Supplementary file 1 [file membranes-13-00672-s001.zip › membranes-2458806-supplementary.pdf]

membranes

MDPI

Supplementary Information

Mesoporous SiC-based photocatalytic membranes and coatings for water treatment

Karla Begonia Cervantes-Diaz, Martin Drobek, Anne Julbe, André Ayral and Julien Cambedouzou *

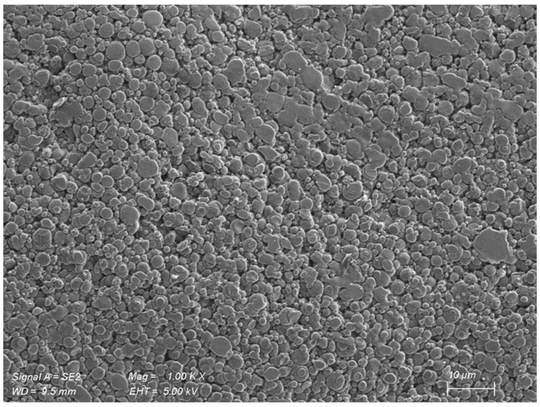

S1. SEM image of the equivalent SiC powder.

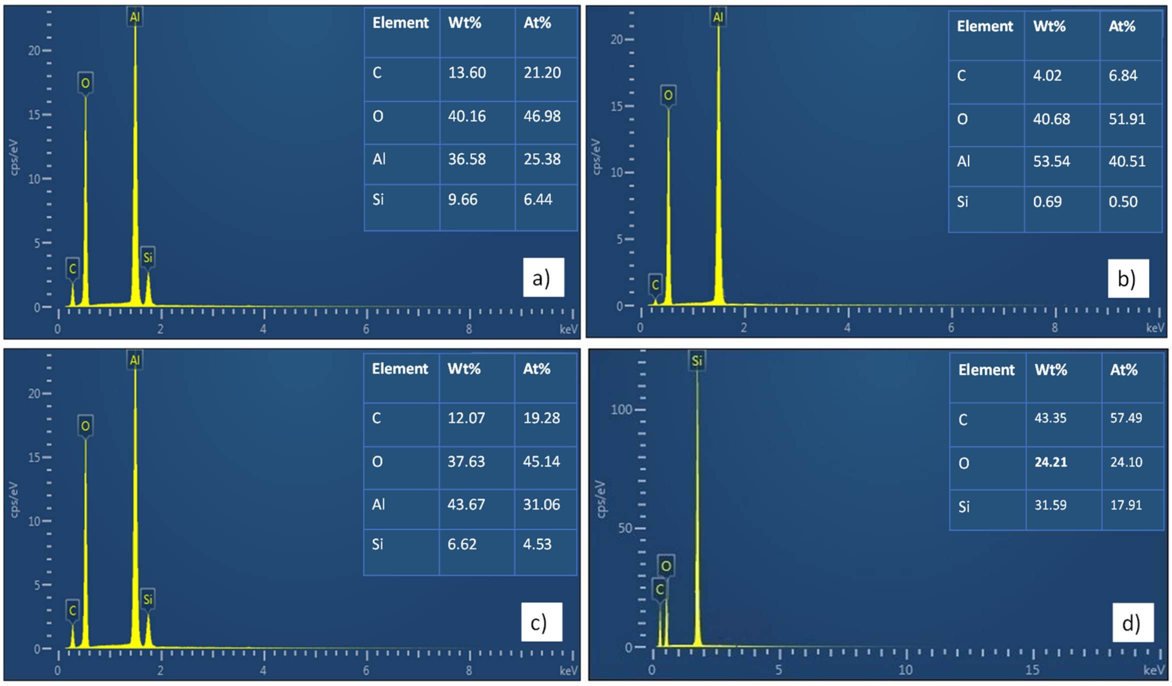

S2. EDX analysis for the SiC membrane (**a**) top surface, (**b**) central zone, (**c**) bottom part of the membrane cross-section. The EDX spectra of the equivalent SiC powder is reported in (**d**) for comparison.

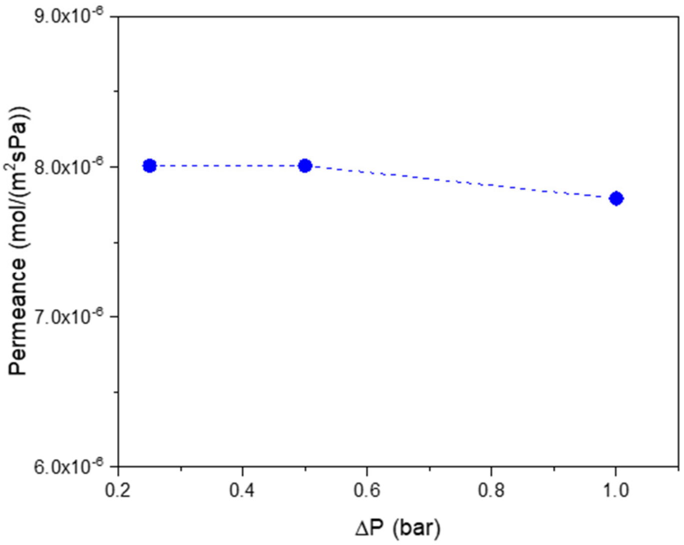

S3. N_2 permeance for SiC-based composite membrane as a function of the applied transmembrane pressure.

S4. Details on Kubelka-Munk equation used for bandgap calculation.

The band gap energy was determined with the Kubelka-Munk function $F(R)$, where the reflectance data can be converted to an absorption coefficient as shown in eq. 1:

$$F(R) = \frac{K}{S} = \frac{(1-R)^2}{2R} \quad (1)$$

with R the reflectance, K the absorption coefficient and S the scattering coefficient. Kubelka-Munk function could be substituted by the absorption coefficient α as in eq. 2 [42,43]:

$$K = 2\alpha = SF(R) \quad (2)$$

Then, from Tauc equation (eq. 3):

$$(\alpha h\upsilon)^{1/n} = A(h\upsilon - E_g) \quad (3)$$

with h the Plank constant, υ the photon frequency, A a proportional constant, E_g the bandgap energy and n determine the transition nature with values of n=1/2 for direct allowed transition, n=3/2 for direct forbidden transition, n=2 for indirect allowed transition, and n=3 for indirect forbidden transition.
Substituting $F(R)$ instead of α gives (eq. 4):

$$(F(R)h\upsilon)^{1/n} = A'(h\upsilon - E_g) \quad (4)$$

with $A' = \frac{2A}{S}$ a constant [45]. Then, the bandgap is calculated by plotting $(F(R)h\upsilon)^{1/n}$ as a function of the photon energy $h\upsilon$ and from the intersection with the x-axis of the linear fit of the region associated with the optical absorption edge [42–44].

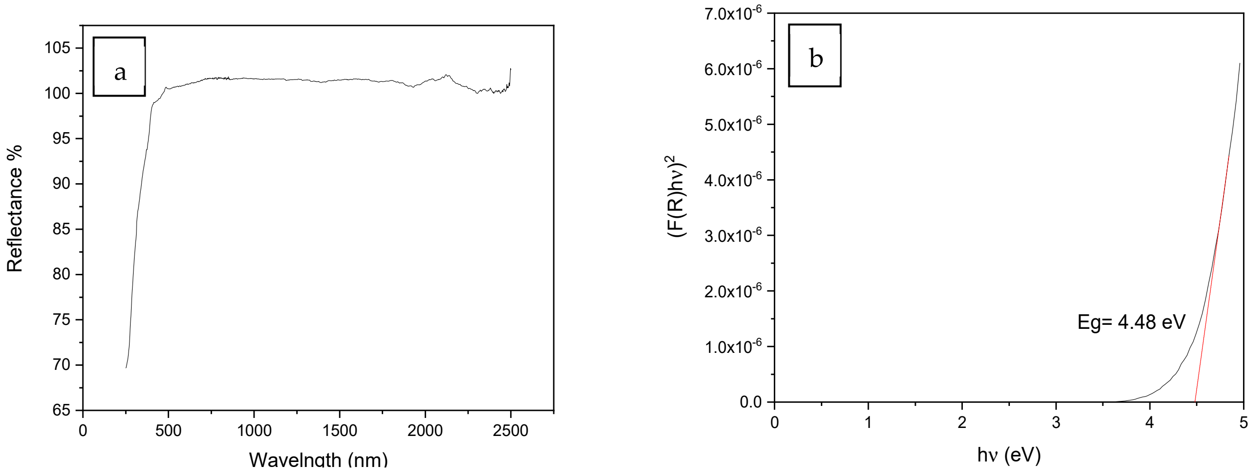

S5. (**a**) Reflectance spectrum and (**b**) calculated bandgap for the pristine α-Al_2O_3 support.

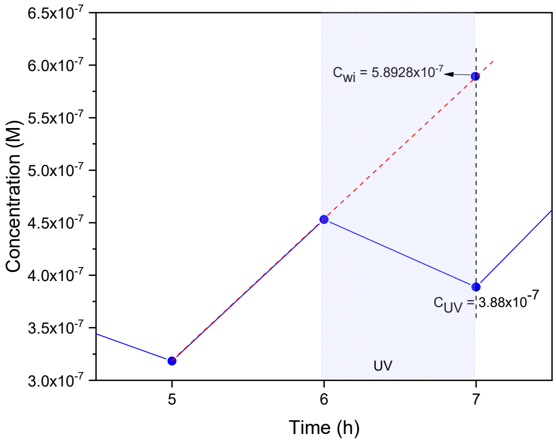

S6. Example of graphical representation used to determine the concentration values for the calculation of the specific degradation rate ẟ.

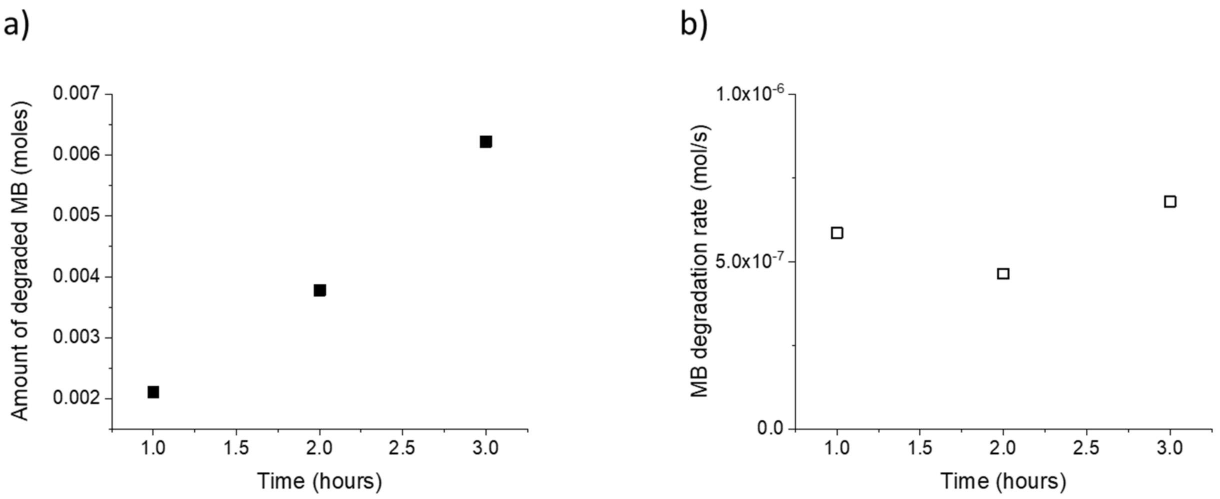

S7. (**a**) Cumulative amount of degraded MB and (**b**) MB abatement rate during photocatalytic experiments in batch mode.
